# Acute hepatitis associated with Q fever in a man in Greece: a case report

**DOI:** 10.1186/1752-1947-1-154

**Published:** 2007-11-27

**Authors:** Magdalini Pape, Andreas Xanthis, Apostolos Hatzitolios, Kalliopi Mandraveli, Christos Savopoulos, Stella Alexiou-Daniel

**Affiliations:** 1 Department of Microbiology, School of Medicine, laboratory of infectious diseases, AHEPA Hospital, Aristotle University of Thessaloniki, Greece; 2 First Medical Propedeutic Department of Internal Medicine, AHEPA Hospital, Aristotle University of Thessaloniki, Greece

## Abstract

Coxiella burnetii is the causative agent of Q fever. Q fever is a worldwide zoonosis that is responsible for various clinical manifestations. However, in Greece hepatitis due to Coxiella is rarely encountered. A case of Q fever associated with hepatitis is reported here. Diagnosis was made by specific serological investigation (enzyme-linked immunosorbent and indirect immunofluorescene assays) for Coxiella burnetii.

## Introduction

Q fever is caused by the obligate intracellular bacterium Coxiella burnetii. The primary reservoirs of infection are farm animals such as cattle, goats and sheep. Pets, including cats, rabbits and dogs, have also been identified as potential sources of human infection. The infected mammals shed the microorganism in urine, feces, milk and especially birth products [1]. The disease can be transmitted mainly through contact with infected animals, inhalation of contaminated aerosols and ingestion of unpasteurized products. Incidents following blood transfusion, skin trauma and sexual contact have been rarely reported. The clinical presentation of Coxiella burnetii is very pleomorfic and non-specific. The infection has two forms, acute and chronic, whereas half of the patients remain asymptomatic.

Among those who are symptomatic the acute form is typically manifested as pneumonia, flu-like syndrome, hepatitis and rarely as Guillain-Barre or lymphadenopathy. Endocarditis is the main clinical form of chronic Q fever and mostly affects patients with underlying valvulopathy. Reports from several places in Europe, such as Great Britain [2], Spain [3], France [4] and Crete, Greece [5] indicate that epidemiological and clinical features of Q fever vary from area to area. Q fever in northern Greece has been rarely reported and may remain underdiagnosed [6].

## Case presentation

In December 2005, a patient aged 22 years was admitted to the emergency department of AHEPA University Hospital of Thessaloniki due to persistent (5 days) high grade of fever (38.5°C) and pharyngalgia. On physical examination no specific clinical signs were present. The initial laboratory tests were normal and chest X ray did not reveal any lung disease. Empiric antibacterial therapy (clarithromycin 500 mg × 2 for 5 days) and non-steroidal anti-inflammatory agent (nimesulid 100 mg × 2 for 3 days) for pyrexia were initiated. During the following week, fever persisted and the patient also developed fatigue, chills, anorexia, headaches, myalgia and skin rash (pink macular lesions of the trunk). When he revisited the emergency department, he was hospitalized for further diagnostic evaluation. The patient had no history of contact with animals, exposure to hepatotoxic agents, like alcohol, drugs, recent history of blood transfusion, or surgical/dental operation.

On clinical examination, jaundice, mild hepatomegaly and skin rash were detected. Chest X ray was found normal and abdominal ultrasound revealed mild hepatomegaly without biliary tract obstruction. Laboratory examinations revealed leukopenia (WBC 2.9 × 10^9^/L), thrombocytopenia (PLT 130 × 10^9^/L), moderate hyperbilirubinemia -mainly direct bilirubin- (T-Bil 3 mg/dL), elevated serum C-reactive protein (2.95 mg/dl) and increased hepatic enzyme levels [ALT: 250 U/L (normal:0–40 U/L), AST: 380 U/L (normal:0–39 U/L), LDH: 900 (normal:240–480 U/L)], whereas cholostatic enzymes (ALP, γ-GT) were found nearly normal.

The patient did not exhibit autoantibodies, including smooth muscle, anticardiolipin, antiphospholipid and antinuclear antibodies. Serologic tests for HIV-1, EBV, CMV, Mycoplasma, Rickettsia, Chlamydia, Bartonella, Parvovirus B19, hepatitis A, B, and C viruses were negative. Q fever was added to the list of differential diagnosis, although exposure to cattle, sheep, goats or consumption of unpasteurized products was not reported. Additionally, a heart ultrasound was performed and pericarditis or myocarditis were excluded.

The diagnosis of acute Q fever was confirmed by serologic methods. Serum samples were tested initially by enzyme-linked immunosorbent assay (ELISA) and its positive result [IgG I (1,1x cutoff), IgG II 41 IU/ml)] was confirmed by indirect immunofluorescene assay (IFA). IgG antibodies were reactive with phase I and II antigens of C. burnetii at titers 1:64 and 1:256 respectively. The patient was administered moxifloxacin 400 mg once a day per os for 14 days. The symptoms resolved within 2 weeks, whereas the levels of hepatic transaminases were mildly elevated [ALT: 55 U/L, AST:63 U/L, T-Bilirubin:1,6 mg/dl]. A convalescent-phase serum sample was obtained 3 weeks later, confirming the initial diagnosis. It was also tested by ELISA [IgG I (1,9x cutoff), IgG II 149 IU/ml)] and IFA [IgG I 1:256, IgG II 1:1024]. During a follow-up visit 3 months after hospitalization, the patient was clinically asymptomatic and had normal hepatic enzymes.

## Discussion

Although described years ago, Q fever is still a poorly understood disease. The clinical manifestations of Q fever may be so variable that the disease is often diagnosed only if it has been systematically considered. Many times, it is diagnosed as a form of atypical pneumonia with or without liver participation, whereas in our case there was no pulmonary disease. Q fever hepatitis has been rarely reported in Greece [7]. Results of this study suggest, however, that acute Q fever should be added to the list of differential diagnosis of patients with fever and elevated serum transaminase levels [8,9], irrespective of the presence of abdominal pain, jaundice and exposure to potentially infected animals.

## Conclusion

Q fever is certainly not the first diagnosis to consider in a patient presenting with fever, rash and constitutional symptoms and as far as we are concerned, it is not routinely tested in most laboratories. In cases with clinical and epidemiological findings compatible with Q fever, coxiella testing should be offered.

## Competing interests

The author(s) declare that they have no competing interests.

## Authors' contributions

MP and A Xanthis are the primary contributing authors. MP is a biopathologist specialist who performed the ELISA tests and AX is the responsible medical internist for the patient. KM is the Associate Director of the Infectious Disease Department of AHEPA Hospital. SA-D is the Professor of Medical Microbiology, CS and A Hatzitolios are Associate Professors in the Medical Department that hospitalized the patient in Aristotle University of Thessaloniki. All author read and approved the subscripted manuscript.

## Consent

Writteninformed patient consent was obtainedfor publication of this case report.
